# Is equine colic seasonal? Novel application of a model based approach

**DOI:** 10.1186/1746-6148-2-27

**Published:** 2006-08-24

**Authors:** Debra C Archer, Gina L Pinchbeck, Christopher J Proudman, Helen E Clough

**Affiliations:** 1 Epidemiology Group, Department of Veterinary Science, University of Liverpool, Leahurst, Neston, Wirral, CH64 7TE, UK

## Abstract

**Background:**

Colic is an important cause of mortality and morbidity in domesticated horses yet many questions about this condition remain to be answered. One such question is: does season have an effect on the occurrence of colic? Time-series analysis provides a rigorous statistical approach to this question but until now, to our knowledge, it has not been used in this context. Traditional time-series modelling approaches have limited applicability in the case of relatively rare diseases, such as specific types of equine colic. In this paper we present a modelling approach that respects the discrete nature of the count data and, using a regression model with a correlated latent variable and one with a linear trend, we explored the seasonality of specific types of colic occurring at a UK referral hospital between January 1995–December 2004.

**Results:**

Six- and twelve-month cyclical patterns were identified for all colics, all medical colics, epiploic foramen entrapment (EFE), equine grass sickness (EGS), surgically treated and large colon displacement/torsion colic groups. A twelve-month cyclical pattern only was seen in the large colon impaction colic group. There was no evidence of any cyclical pattern in the pedunculated lipoma group. These results were consistent irrespective of whether we were using a model including latent correlation or trend. Problems were encountered in attempting to include both trend and latent serial dependence in models simultaneously; this is likely to be a consequence of a lack of power to separate these two effects in the presence of small counts, yet in reality the underlying physical effect is likely to be a combination of both.

**Conclusion:**

The use of a regression model with either an autocorrelated latent variable or a linear trend has allowed us to establish formally a seasonal component to certain types of colic presented to a UK referral hospital over a 10 year period. These patterns appeared to coincide with either times of managemental change or periods when horses are more likely to be intensively managed. Further studies are required to identify the determinants of the observed seasonality. Importantly, this type of regression model has applications beyond the study of equine colic and it may be useful in the investigation of seasonal patterns in other, relatively rare, conditions in all species.

## Background

Analysis of temporal patterns in data (i.e. data that arises over time) constitutes an important area of statistics, with applications in a wide range of fields from economics to engineering [1]. Consistent seasonal patterns in disease suggest the possibility of predictable behaviour, and in human medicine these have assisted rational planning of hospital resources in addition to providing clues regarding disease aetiology. The latter aspect is important in stimulating research to further the understanding of disease causality. Time-series analysis has been used in the human medical field to investigate a number of non-infectious conditions including asthma and aortic aneurysms [2] and in veterinary epidemiology to investigate patterns in infectious diseases [3-6]. However these statistical methods have received relatively little attention in the field of non-infectious veterinary diseases and, to our knowledge, have not previously been reported in the investigation of colic in the horse.

Colic is an important cause of mortality and morbidity in domesticated horses and has a complex, multifactorial nature [7-10]. Many questions about this condition remain to be answered including the effect of season on the occurrence of colic. Knowledge of a seasonal pattern (or indeed lack of evidence of a seasonal pattern) in the incidence of colic within a population could assist identification of risk-factors for this disease. Such information could be used to devise preventative strategies, such as altered management practices, to potentially reduce its occurrence. Increased incidence of colic has been identified in certain months of the year in several different equine populations [8,11-14] but the association between season and colic is unclear. This may, in part, be attributable to limitations in the statistical approaches that have previously been used to address this issue [15].

Many standard statistical approaches are built upon the assumption that observations are mutually independent. This assumption is likely to be inappropriate in the case of colic since many factors may be interdependent; observations in adjacent months might be more similar than those which occur months apart due to, for example, similarities in feed types and duration of stabling. Time-series methods provide a valid means of investigating seasonal patterns in colic. Traditional approaches, such as the Auto-regressive Integrated Moving Average (ARIMA) of Box and Jenkins [16] offer a number of possibilities. However, this approach requires the number of observations at each time of interest to be large for the Normal distribution-based assumptions upon which it is based to remain valid. This method would not be suitable for diseases, such as admissions of colic cases to a hospital, in which the counts per month are relatively small (i.e. typically less than 30). In the latter situation, it is necessary to use a modelling approach that respects the discrete nature of the count data. One possibility lies in the use of a Poisson distribution to model count data within a framework broadly analogous to that of generalised linear modelling [17].

The aim of this study was to determine if there was any evidence of seasonality in horses presented to a UK referral hospital with particular types of colic. Using a Bayesian approach, we fitted a regression model which incorporated autocorrelation as a latent variable, to reflect the fact that, having taken account of seasonality and trend, any remaining serial dependence may operate over a shorter temporal scale and is likely to represent unmeasured influential covariates which themselves vary over time. In addition we fitted a model without latent correlation but with a linear trend. Based on current evidence in the literature, our *a priori *hypotheses were that equine grass sickness (EGS) and epiploic foramen entrapments (EFE) would demonstrate seasonality but that intestinal obstruction by pedunculated lipomas would be a random event without any evidence of seasonality. It was unclear if a seasonal effect would be seen in the other colic groups.

## Results

### Exploratory data analysis

**Figure 1:**
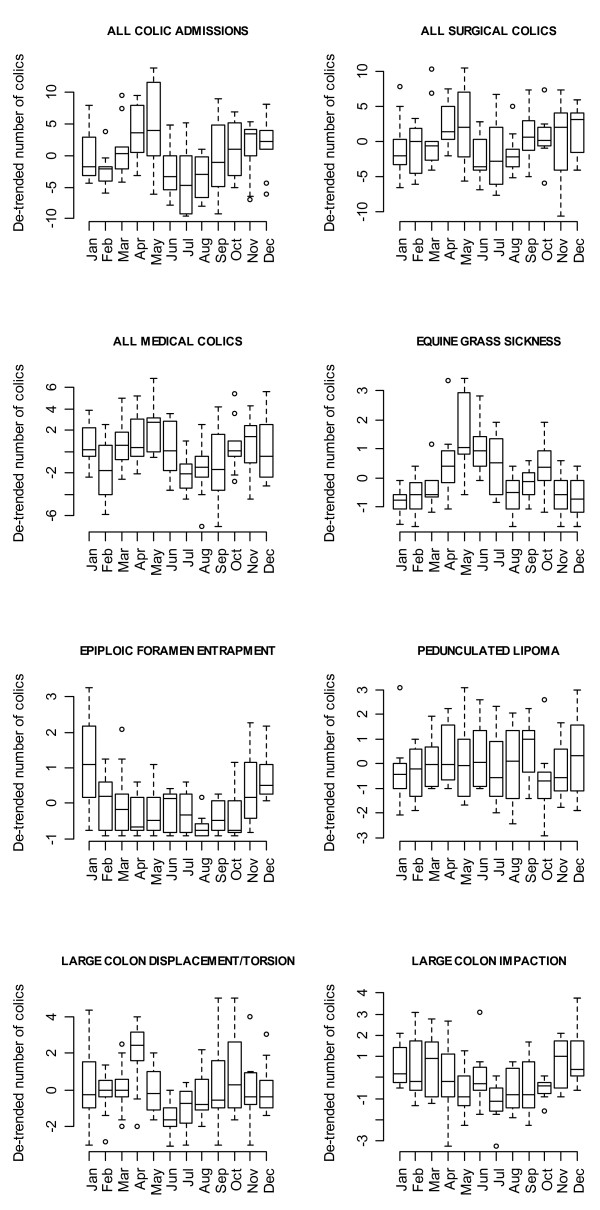
Boxplots of de-trended (annual average subtracted) colic admissions by month for each colic admitted to a UK referral hospital between January 1995 – December 2004.

The total numbers of colic cases for each diagnostic category are shown in Table 1 and boxplots of detrended colic admissions by month for each colic group are presented in Figure 1. Total admissions of all colic cases to the hospital appeared to peak in the months of April/May and again in October/November/December. A similar pattern was also evident in the medically and surgically treated colic groups. There was a clear seasonal effect for EGS, with a pronounced peak in May and a suggestion of a secondary peak in October. Cases of EFE appeared to peak in the months of December/January. There did appear to be a possible seasonal component to cases of large colon displacements and torsions, with peaks in the spring and autumn months, whereas primary large colon impaction colics appeared to peak over the autumn and winter months. There was no graphical evidence of a seasonal effect in cases of pedunculated lipoma.

### Regression model with seasonal components, trend and an autocorrelated latent variable

**Figure 2:**
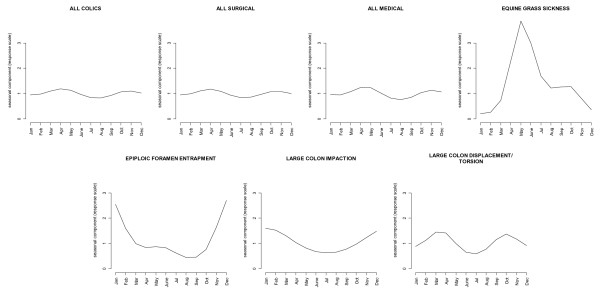
**Estimate of model's seasonal component for each colic type**. For each colic type an estimate of the model's seasonal component was extracted using the posterior mean of the parameter associated with each of the sine and cosine terms based on the frequencies detected for each group in Table 2. With the exception of the large colon impaction group (12 month cycles only) all models incorporated 12- and 6-monthly cycles.

The posterior distribution summaries for each colic type are presented in Table 2. With the exception of lipoma for which our preliminary assessment of no evidence of a seasonal pattern was confirmed, the only colic for which a model with twelve-monthly cycles only appeared superior to a model with 12 and 6-month cycles was large colon displacement/torsion. Twelve and 6-monthly cyclical components were identified for EGS, large colon impaction and EFE colics. Weaker 12 and 6-monthly cycles were evident in the all colics, all medically treated colics and all surgical colics groups. The weaker signal in the latter three is justified by the fact that these represent combinations of colics of different types, each of which has their own distinct seasonal profile. Despite this level of aggregation a small seasonal profile emerges. Note that although the credible intervals for sine and cosine terms representing 12-month cyclical components for all colics, surgical colics and medical colics do not strictly exclude 0, in each case their extremity is very close to 0 and so we retained these terms. Unsurprisingly, more convincing segregation between models upon the basis of the DIC statistic was possible in the cases where larger data sets were available for analysis (all colics, all surgical colics, all medical colics and large colon displacements), and we should interpret the findings in the cases where counts per month are small more cautiously. Estimates of the seasonal component for the "best" model and its relationship to the month of the year for each colic type are shown in Figure 2.

The inclusion of trend and serial correlation together in models of this nature where the number of cases observed at a particular time point is small is potentially problematic, as it may prove difficult to separate positive serial dependence and trend. Indeed, if positive trend exists and there may be positive serial correlation, parameters in the model are potentially highly correlated and the MCMC algorithm struggles in the presence of low counts. As expected there were problems with convergence for many of the models including both terms; we therefore do not include the DICs from models incorporating latent serial correlation together with a linear trend in Table 3 because they are likely to be unreliable.

Models either without trend/with latent serial correlation or with trend/without latent serial correlation, provided better convergence of the MCMC algorithm. For the same data set we find situations where a model with latent serial correlation and 12- and 6-month cycles but no trend term is selected as optimal by DIC comparison (Table 3), whereas in the case where serial dependence is excluded, a model with those same seasonal components and a positive trend is selected (Table 4). With the exception of EFE for which no evidence of trend emerges, for each of these latter models the trend term is of the order of 0.005 (standard deviation of the order of 0.002). More compelling evidence of an increasing trend over time occurs in the cases where sample sizes are larger.

In the model incorporating latent serial correlation but no trend, it is interesting that although the parameter which controls the dependence (α) does not have a marked effect on the model (as judged by the fact that the credible interval contains 0) the posterior mean for α in all cases, though small, is positive. Whilst we must be cautious concerning over-interpretation of this finding in the presence of large uncertainty, a small but positive effect may represent positive serial correlation, or it could in part be measuring the increasing trend which we were unable to include simultaneously for statistical reasons. (Note that, whilst comparisons within Tables are valid, comparisons between DICs presented in Table 3 and Table 4 cannot be drawn, as they represent different classes of models, one with and one without a latent correlation structure).

For our purposes, given that our primary interest concerns seasonality, whether we included latent serial correlation or trend, the estimates of the seasonal components were broadly similar across models and this renders our findings regarding seasonality robust in the presence of these largely statistical effects.

## Discussion

The aim of the present study was to investigate the seasonality of different types of colic presented at a UK equine referral hospital. Cohen [15] stated the need for new statistical or epidemiological models that could address deficiencies in our knowledge regarding equine colic. This model provides a useful means of investigating temporal patterns in equine colic, and to our knowledge, this is the first report that uses time-series methods of analysis to explore seasonal patterns in equine colic.

Two studies in the UK have described an apparent peak in cases of colic of any cause in spring and autumn months [11,13]. In the present study, similar patterns were evident in the all colic and all medically or surgically treated colic groups with small peaks evident around the months of March/April and October/November. Hillyer et al. [13] suggested that the seasonal pattern of colic in the racehorse population under investigation in their study may have been associated with stage of training or level of activity. Increased risk of colic has been identified following change in diet and stabling in the preceding 2 weeks [18,19] and following decreased exposure to pasture [20]. Therefore, these patterns of colic may not be surprising given that, at these times of the year in the UK, changes in management practices such as turnout, stabling and exercise are more likely to occur.

This modelling approach confirmed our hypothesis that EGS would exhibit seasonality, as demonstrated by other workers using different approaches. Although EGS may occur at any time of the year, the peak incidence of this condition in the UK is reported in the months of spring and summer, and the month of May in particular [21,22]. In the present study, EGS exhibited significant 12- and 6- month cyclical components, cases peaking in the month of May with a secondary less pronounced peak in the month of October. Risk factors for EGS that have been identified in epidemiological studies previously include increased risk associated with change of field in the previous 2 weeks [22], non-feeding of hay or haylage and change of feed type or quantity 14 days prior to disease [23]. The seasonal pattern of EGS identified in the present study coincides with months of the year that may be associated with change in grazing practices and feed types in the UK.

Use of this model also confirmed our hypothesis that EFE would exhibit seasonality. Using data arising over a 10 year period at the same hospital (1991–2001), multivariable modelling confirmed that EFE was consistently more prevalent in the months of December, January and February [24]. There was a suggestion of a seasonal pattern of distribution for each year studied but, using traditional methods of analysis, we were unable to confirm this statistically. The results from the present study revealed 6- and 12- month cyclical components to cases of EFE presented at this hospital; the main peak occurred in the months of November, December and January with a secondary, less pronounced peak in the months of April, May and June. In Germany, Scheideman [25] reported that although EFE cases were seen throughout the year, a marked increase in cases was evident during the period between December and April. The seasonality of EFE may reflect changes in stabling, turnout, exercise and feeding practices common to these times of the year; these are currently under investigation in a prospective study.

The large colon impaction colic group exhibited 12 month cyclicity, with an increasing number of cases identified in the autumn and winter months (peak December/January) decreasing over the spring months with the lowest incidence over the months of July and August. A slightly different cyclical pattern was identified in the large colon displacement/torsion colic group with peak incidence in the months of Spring and Autumn, similar to that seen in the all colic and all medically or surgically treated colic groups. Hillyer et al. [26] identified a number of factors associated with increased risk of simple colonic obstruction and distension colic (defined as primary large colon impactions and simple large colon displacements). These included an increasing number of hours spent in a stable, recent change in a regular exercise programme and stabling for 24 hours per day. These factors may explain the reduced incidence of colic of either type evident in the months of June, July and August when horses, in general, are less likely to be stabled for prolonged periods in the UK. Many factors have been associated with large colon impactions including acute decrease in exercise or cessation of daily turnout [27] and feeding of coarse roughage [28]. These factors may, in part, account for the increased incidence of this colic type coinciding with months of the year when cold, wet weather is more likely to occur in the UK. Under these conditions horses are more likely to be housed and to be given more supplementary roughage (i.e. hay/haylage in addition to grass). Large colon torsion has been associated with mares in the periparturient period [28] which might explain the increased prevalence of this colic type between the months of January and May; however brood mares comprise a relatively small component of this hospital's caseload.

Obstruction of intestine by pedunculated lipomas in theory should be a random event, and this model confirmed our *a priori *hypothesis that no seasonal component to this condition would be identified.

We have alluded to the difficulties in detecting serial dependence in the presence of trend when samples are small. With larger samples it might be possible to separate more conclusively trend and latent serial dependence and further research using larger samples sizes is warranted.

Considering first the possible interpretation of latent serial correlation in the context of colic, we take EGS as an example. The role of *Clostridium botulinum *in EGS has received renewed interest [29]. Taking the assumption that *C. botulinum *does play a role in the aetiology of this specific cause of colic as a working hypothesis, it would seem plausible that the levels of the pathogen in the environment will be temporally structured so that they are similar in proximate months and less similar in months which are far apart, irrespective of the seasonal effect. Using space-time K-function analysis, French et al. [30] demonstrated strong evidence of space-time clustering of this disease, particularly within the first 10 km and 20 days of a case, which would support the latter idea. Similarly, feed types and amounts, periods of stabling and turnout are more likely to be similar in proximate months.

Considering now the interpretation of a positive linear trend which was evident in all models excepting that for EFE not including latent correlation, knowledge of continued improvements in the medical and surgical management of colic and resultant increased success rates following treatment [31] may have positively influenced referring vets and owners making them more willing to undertake referral. This trend may also reflect increased levels of insurance in the hospital referral population, making surgical correction or intensive medical treatment an option when previously it may not have been affordable. In the case of colic due to intestinal obstruction by a peduncluated lipoma, which most frequently occurs in older ponies and horses [32-34], a combination of affordability and knowledge that surgical success rates following treatment of this condition are comparable to, or in some cases better than, other surgical lesions in younger horses [35] may account for this annual trend. Alternatively, there may simply be a greater number of older ponies or horses in the general equine population [36]. It was also interesting to note that an annual trend was not evident in cases of EFE admitted to the hospital. This finding may be due to insufficient power to detect a marked effect based on the relatively small numbers of EFE in this series.

Weather-related factors have not been shown to be statistically significant in relation to colic using traditional methods of analysis, despite many anecdotal reports to the contrary [11,37-39]. It is important to consider that climatic conditions may be confounded by other factors. For example, extreme weather conditions may result in altered management practices such as reduced level of horse activity [40]. Nevertheless, identification of any weather-related patterns associated with colic may assist identification of causal factors. Time-series analysis provides a more elegant and valid means of studying seasonal patterns to colic and may also provide a more appropriate means of investigating associations between weather patterns and disease [5].

A number of approaches may be used to investigate temporal patterns in data and, when choosing the most suitable method, it is important to recognise that different types of dependence which are context-specific may occur. First, the number of events in month *t *might explicitly depend upon the number of events in month *t*-1 e.g. if one is considering the evolution of an infectious disease which propagates by direct contact between infected individuals. This type of dependence is described as "observation driven" [41]. Secondly, the counts in month *t *and month *t*-1 might be independent, conditional upon some latent process which is temporally structured and contains serial correlation. For example, the number of individuals suffering from hypothermia might be influenced by climatic conditions, which themselves vary with time, and are likely to be autocorrelated i.e. the weather in month *t *is likely to be in some way similar to the weather in month *t*-1. Here, dependence (and subsequent models) is described as "parameter driven" [42]. The two dependence assumptions are qualitatively different and require different modelling approaches. There is little reason to suppose that the number of colic cases admitted to a hospital facility in month *t *is directly influenced by the number in the previous month (*t*-1). Instead, it seems more plausible that there may be some underlying, unmeasured (or indeed immeasurable) process which has a direct influence on the monthly counts. It is our belief that the parameter driven approach is likely to be most relevant to data pertaining to colic in the horse and is the basis upon which the model was chosen.

An important issue in Markov Chain Monte Carlo (MCMC) based analysis is that of convergence of the Markov Chains and whether the samples being generated are from the true posterior distribution under the model framework. In order to test this, we ran two chains simultaneously using differing starting values, and found that in each case the posterior summaries obtained were analogous. In addition, we examined the R^
 MathType@MTEF@5@5@+=feaafiart1ev1aaatCvAUfKttLearuWrP9MDH5MBPbIqV92AaeXatLxBI9gBaebbnrfifHhDYfgasaacH8akY=wiFfYdH8Gipec8Eeeu0xXdbba9frFj0=OqFfea0dXdd9vqai=hGuQ8kuc9pgc9s8qqaq=dirpe0xb9q8qiLsFr0=vr0=vr0dc8meaabaqaciaacaGaaeqabaqabeGadaaakeaacuWGsbGugaqcaaaa@2DE9@ statistic (the "potential scale reduction factor") provided by WinBUGS and found that in all cases barring the models which attempted to incorporate both trend and latent correlation this was very close to 1.

A further issue in Bayesian analysis concerns the sensitivity of the resultant posterior distribution to the choice of prior distribution. Given that, for all parameters, we have selected vague priors we do not believe this to be an issue here; in addition, although the counts at each time point were relatively small, the length of each series was large (n = 120 in all but one case where n = 119) so we would expect the data to dominate.

The issue of determining a suitable autocorrelation structure for the error term in these models is also important. There exists only a single series of data, in contrast with a longitudinal data set for which we can gain knowledge about the autocorrelation structure by exploiting the replication in the data [43]. Our selection of a latent variable including only first-order correlation (correlation with the previous time point) is rather arbitrary, but seems reasonable on scientific grounds in that there may be environmental factors which are very similar in proximate months. It would be possible within this modelling framework to incorporate more complex error structures, for example, allowing dependence on even earlier time points. It is likely, however, that with the small counts available longer-term effects of this nature could not be detected.

The exact gastrointestinal dysfunction or lesion is unknown in many cases of colic that occur within the general equine population [10,11,20]. It is important to recognise that data based on colic cases presented to a referral hospital represent only a small proportion of all colic cases occurring within a geographical location: such a population is biased towards horses with lesions requiring surgical correction or more intensive medical treatment, and whose owners are willing to undertake referral. In addition, studies investigating specifically diagnosed cases of colic would include only a minority of cases seen in the general population [8]. However such studies are necessary due to the fact that risk-factors and patterns of disease may be different for various types of colic, and investigation of colic of any cause may miss some of these [44]. The colic types investigated in the present study also represent the more severe forms of the disease i.e. those which do not resolve spontaneously or following simple medical treatment, making the investigation of causality and potential prevention of relatively greater importance. It is unlikely that there would be any effect of season on the referral of colic cases to the clinic.

The models produced in this paper are biologically plausible and provide useful information on the temporal patterns of different colic types. This work demonstrates in principle how standard and non-standard Poisson regression-based approaches can be used in other veterinary applications where disease incidence is relatively rare. These results also provide an insight into the aetiology of different colic types admitted to a UK referral hospital. There is a suggestion of increased admissions of certain colic types at times of managemental change (surgically and/or medically treated colics, large colon displacements/torsions and EGS) and during periods of intensive management (months of the year when horses are more likely to be stabled or stabled for longer periods of time) e.g. EFE and large colon impaction. These results are based on the findings from a single UK referral equine hospital; further studies are required to determine the relationship between season and colic incidence in other geographical locations using hospital and non-hospital based populations.

## Conclusion

We have used a regression model which has the flexibility to incorporate latent serial correlation to explore the seasonal prevalence of different colic types presented at a UK equine referral hospital. This is a novel statistical approach in the field of equine colic research and it has enabled us to confirm a seasonal pattern for equine grass sickness, as demonstrated by other workers using different methods of analysis, and to formally establish the existence of a marked seasonal effect in cases of epiploic foramen entrapment. In addition, a seasonal pattern was evident to admissions of all colic types, all surgical and medical colics and in cases of large colon impaction and large colon displacement/volvulus. Use of this model confirmed that intestinal obstruction by pedunculated lipomas showed no seasonal effect. Knowledge of the seasonal associations with certain types of colic is consistent with an aetiological role for managemental change and periods of intense management such as prolonged stabling. Further studies are required to identify the determinants of the observed seasonality. This type of regression model has applications beyond the study of equine colic and it may be useful in the investigation of seasonal patterns in other, relatively rare, conditions in all species.

## Methods

### Colic data

All cases of colic admitted to the Philip Leverhulme Equine Hospital, University of Liverpool between 1^st ^January 1995 and 31^st ^December 2004 were reviewed retrospectively. The numbers of colic cases occurring in each of the 120 months under investigation were recorded and aggregated as counts per month in the groups defined in Table 1.

### Exploratory data analysis

For each colic type, the effect of increasing yearly case numbers was removed (de-trended) by subtracting an annual average to create a residual [45]. A box plot of these residuals by month was then generated. This allowed us to search for preliminary descriptive evidence of seasonality without the data being complicated by the presence of an annual trend (defined as an increase/decrease in the number of colic cases admitted over time for each 12 month period).

### Regression model

Our chosen model for incorporating latent correlation was similar to the generalised linear model with Poisson response and logarithmic link function, which is commonly used to model independent count data [17] but has an added level of complexity in that dependence between observations in the series is explicitly incorporated via a latent variable. This is an example of a Bayesian Hierarchical model (see, for example [46]). This approach allows us, having accounted for seasonality and trend, to determine whether any correlation between observations at successive time points, over a shorter scale than that indicated by cycles or trend, remains. Having accounted for these factors, we can then determine whether observations in two successive months are more (or less) similar than we might expect by chance.

The most general model incorporating cycles at both 6 and 12-month frequencies is as follows: Let *N*_t _be the number of admissions in month t, and *t *indicate annual trend. The harmonic components at 6- and 12-month frequencies are used to represent the seasonal components, and α represent the dependence between latent variables in successive months. From an inferential point of view our interest concerns whether the 95% credible interval for α contains 0, which equates to no evidence of latent serial correlation.

*N*_*t *_~ Poisson(μ_*t*_)

log(μ_*t*_) = β_0 _+ β_1 _sin⁡(2πt12)
 MathType@MTEF@5@5@+=feaafiart1ev1aaatCvAUfKttLearuWrP9MDH5MBPbIqV92AaeXatLxBI9gBaebbnrfifHhDYfgasaacH8akY=wiFfYdH8Gipec8Eeeu0xXdbba9frFj0=OqFfea0dXdd9vqai=hGuQ8kuc9pgc9s8qqaq=dirpe0xb9q8qiLsFr0=vr0=vr0dc8meaabaqaciaacaGaaeqabaqabeGadaaakeaacyGGZbWCcqGGPbqAcqGGUbGBdaqadaqaamaalaaabaGaeGOmaidcciGae8hWdaNaemiDaqhabaGaeGymaeJaeGOmaidaaaGaayjkaiaawMcaaaaa@387C@ + β_2 _cos⁡(2πt12)
 MathType@MTEF@5@5@+=feaafiart1ev1aaatCvAUfKttLearuWrP9MDH5MBPbIqV92AaeXatLxBI9gBaebbnrfifHhDYfgasaacH8akY=wiFfYdH8Gipec8Eeeu0xXdbba9frFj0=OqFfea0dXdd9vqai=hGuQ8kuc9pgc9s8qqaq=dirpe0xb9q8qiLsFr0=vr0=vr0dc8meaabaqaciaacaGaaeqabaqabeGadaaakeaacyGGJbWycqGGVbWBcqGGZbWCdaqadaqaamaalaaabaGaeGOmaidcciGae8hWdaNaemiDaqhabaGaeGymaeJaeGOmaidaaaGaayjkaiaawMcaaaaa@3872@ + β_3 _sin⁡(2πt6)
 MathType@MTEF@5@5@+=feaafiart1ev1aaatCvAUfKttLearuWrP9MDH5MBPbIqV92AaeXatLxBI9gBaebbnrfifHhDYfgasaacH8akY=wiFfYdH8Gipec8Eeeu0xXdbba9frFj0=OqFfea0dXdd9vqai=hGuQ8kuc9pgc9s8qqaq=dirpe0xb9q8qiLsFr0=vr0=vr0dc8meaabaqaciaacaGaaeqabaqabeGadaaakeaacyGGZbWCcqGGPbqAcqGGUbGBdaqadaqaamaalaaabaGaeGOmaidcciGae8hWdaNaemiDaqhabaGaeGOnaydaaaGaayjkaiaawMcaaaaa@3794@ + β_4 _cos⁡(2πt6)
 MathType@MTEF@5@5@+=feaafiart1ev1aaatCvAUfKttLearuWrP9MDH5MBPbIqV92AaeXatLxBI9gBaebbnrfifHhDYfgasaacH8akY=wiFfYdH8Gipec8Eeeu0xXdbba9frFj0=OqFfea0dXdd9vqai=hGuQ8kuc9pgc9s8qqaq=dirpe0xb9q8qiLsFr0=vr0=vr0dc8meaabaqaciaacaGaaeqabaqabeGadaaakeaacyGGJbWycqGGVbWBcqGGZbWCdaqadaqaamaalaaabaGaeGOmaidcciGae8hWdaNaemiDaqhabaGaeGOnaydaaaGaayjkaiaawMcaaaaa@378A@ + β_5_*t *+ *e*_*t*_

*e*_*t *_~ *N*(μ_*t*_,σ_τ_^2^

μ_*t *_= α + *e*_*t*-1_

The model detailed above treats the unobserved variables as a latent, temporally varying process (here autoregressive of order 1 so that the latent variable in the current month is allowed to depend via a Normal distribution on the equivalent latent variable in the previous month; in principle in its most general form the structure could be of order *q *where *q *≥ 1).

The model was fitted within a Bayesian framework as described in [47] using Markov Chain Monte Carlo (MCMC) methods within the software package WinBugs [48] in combination with the R library "R2WinBUGS" [49]. A 'burn-in' of 20,000 iterations was used and a sample of 100,000 realisations from the posterior distribution for each parameter was produced. The output chain for each parameter was thinned to every 10^th ^observation to reduce correlation between samples in the posterior distribution. Vague prior distributions were adopted for each of the β parameters (reflecting a lack of prior belief concerning parameter values), and the prior distribution for α was Uniform on [-1, 1] (although we believe *a priori *that any latent dependence in models for data of this kind is likely to be positive, bounding the parameter in this way allows us to examine the evidence in favour of serial dependence being present via a 95% credible interval for α which excludes 0). Markov chain convergence was assessed by comparing two chains from divergent starting values and comparing traces, and in addition examining the R^
 MathType@MTEF@5@5@+=feaafiart1ev1aaatCvAUfKttLearuWrP9MDH5MBPbIqV92AaeXatLxBI9gBaebbnrfifHhDYfgasaacH8akY=wiFfYdH8Gipec8Eeeu0xXdbba9frFj0=OqFfea0dXdd9vqai=hGuQ8kuc9pgc9s8qqaq=dirpe0xb9q8qiLsFr0=vr0=vr0dc8meaabaqaciaacaGaaeqabaqabeGadaaakeaacuWGsbGugaqcaaaa@2DE9@ statistic provided by WinBUGS which is the "potential scale reduction factor" and for a convergent chain approaches the value 1. Final inference was therefore based upon 16,000 draws (from the two chains judged to be in equilibrium) from the posterior distribution for each parameter. In the case where the 95% credible interval for the sine component at a given frequency excluded 0 but the cosine component did not, or vice versa, both terms were retained due to the fact that the sine and cosine terms together uniquely determine the location and scale of the cycle. Analogous models were compared using the Deviance Information Criterion (DIC) [50] which we present in Tables 3 and 4. The DIC penalises models which are over-complex so that a "good" model represents a balance between plausible explanation of the data and model parsimony; in broad terms, the smaller the DIC, the better the model. In each case, we select as optimal the model which both carries the smallest DIC value and is the simplest.

Within each selected "best" model for each colic, the posterior mean, posterior standard deviation and 95% credible interval for each parameter are given in Table 2. We only report in full parameter estimates for the model with serial dependence and without trend; as we have discussed the estimates of seasonal components in the models with trend but no serial dependence are identical save for sampling variation induced by the MCMC algorithm. Within a Bayesian framework we cannot make statements about the "statistical significance" of parameter estimates as the common concept of a *p*-value and associated concepts of statistical significance are founded upon frequentist, rather than Bayesian, arguments. Instead, as an initial screen, we judged those parameters for which the standard deviation was smaller than half of the mean to have a marked effect on the outcome of interest (mean number of colic cases observed). We also reported the posterior 95% credible interval: an equivalent approach in this case involves identifying parameters for which this interval does not contain the value 0.

For each colic type, an estimate of the model's seasonal component was calculated by exponentiating from the chosen "best" model the sum of the posterior means of the seasonal components on the log scale, thus representing a multiplicative term in a model for the original observations. This enabled us to produce a graphical representation of the cyclical patterns in each group in relation to months of the year (Figure 2).

## Authors' contributions

DA designed the study, acquired the data and drafted the manuscript. HC participated in design of the study, performed the statistical modelling and drafted the manuscript. GP conceived the study, participated in its design and critically reviewed the manuscript. CP compiled the data, participated in the design of the study and critically reviewed the manuscript. All authors have read and approved the final manuscript.

## Figures and Tables

**Table 1:** Colic categories, case definitions and number of cases in each category admitted to the PLEH between January 1^st ^1995 and 31^st ^December 2004

**Colic category**	**Case definition**	**Total number**
All Colics	All confirmed cases of colic admitted to the hospital	2580
All Surgical Colics	Colic cases with surgical lesions confirmed at exploratory laparotomy or post-mortem examination	1612
All Medical Colics	All colic cases that resolved with medical treatment only	968
Pedunculated Lipoma	Obstruction of small intestine by a pedunculated lipoma diagnosed at exploratory laparotomy or post-mortem examination	231
Epiploic Foramen Entrapment	Entrapment of the small intestine in the epiploic foramen diagnosed at exploratory laparotomy or post-mortem examination	92
Equine Grass Sickness	Equine grass sickeness cases confirmed by histological examination of the ileum	109
Large colon displacements or torsions	Displacement or torsion of the large colon diagnosed by rectal examination, clinical signs and response to treatment; treated either surgically or medically or diagnosed at post-mortem examination	435
Large colon impactions	Primary large colon impactions confirmed by rectal examination and response to treatment (medically treated group) or at exploratory laparotomy	214

**Table 2:** Parameter estimates from the regression models for each colic type.

**Colic type**	**Parameter**	**Posterior Mean**	**Posterior Standard Deviation**	**95% Credible Interval**
All Colics	Intercept	2.849	0.966	1.059, 4.737
	S_12_	0.082	0.043	-0.002, 0.167
	C_12_	0.029	0.043	-0.055, 0.113
	S_6_	-0.132	0.033	-0.196, -0.067
	C_6_	-0.007	0.033	-0.071, 0.058
	α	0.005	0.012	-0.018, 0.029
All Surgical	Intercept	2.159	1.089	-0.017, 4.156
	S_12_	0.065	0.054	-0.042, 0.173
	C_12_	0.034	0.055	-0.073, 0.142
	S_6_	-0.114	0.042	-0.196, -0.032
	C_6_	-0.037	0.041	-0.119, 0.044
	α	0.007	0.015	-0.024, 0.037
All Medical	Intercept	2.218	1.035	0.271, 4.225
	S_12_	0.117	0.061	-0.001, 0.237
	C_12_	0.021	0.059	-0.095, 0.136
	S_6_	-0.167	0.051	-0.267, -0.067
	C_6_	0.044	0.049	-0.054, 0.140
	α	0.004	0.014	-0.023, 0.031
Equine Grass Sickness	Intercept	-1.430	1.278	-3.750, 1.244
	S_12_	-0.275	0.190	-0.655, 0.093
	C_12_	-1.060	0.206	-1.481, -0.673
	S_6_	-0.638	0.172	-0.980, -0.306
	C_6_	0.041	0.163	-0.277, 0.357
	α	0.006	0.024	-0.042, 0.054
Epiploic Foramen Entrapment	Intercept	-0.698	1.029	-2.710, 1.456
	S_12_	0.396	0.199	0.013, 0.794
	C_12_	0.590	0.168	0.271, 0.929
	S_6_	0.028	0.167	-0.302, 0.356
	C_6_	0.404	0.169	0.077, 0.736
	α	0.002	0.020	-0.038, 0.041
Pedunculated Lipoma	Intercept	-0.253	1.123	-2.489, 1.872
	α	0.010	0.019	-0.028, 0.049
Large Colon Impaction	Intercept	0.057	0.957	-1.643, 1.999
	S_12_	0.265	0.118	0.033, 0.497
	C_12_	0.389	0.118	0.162, 0.622
	α	0.005	0.021	-0.038, 0.046
Large Colon Displacement/Torsion	Intercept	-0.275	1.112	-2.388, 2.065
	S_12_	0.116	0.101	-0.084, 0.315
	C_12_	0.166	0.110	-0.049, 0.383
	S_6_	-0.234	0.090	-0.410, -0.058
	C_6_	-0.256	0.090	-0.433, -0.080
	α	0.005	0.022	-0.039, 0.049

For compactness, S_12_ = sin⁡(2πt12)
 MathType@MTEF@5@5@+=feaafiart1ev1aaatCvAUfKttLearuWrP9MDH5MBPbIqV92AaeXatLxBI9gBaebbnrfifHhDYfgasaacH8akY=wiFfYdH8Gipec8Eeeu0xXdbba9frFj0=OqFfea0dXdd9vqai=hGuQ8kuc9pgc9s8qqaq=dirpe0xb9q8qiLsFr0=vr0=vr0dc8meaabaqaciaacaGaaeqabaqabeGadaaakeaacyGGZbWCcqGGPbqAcqGGUbGBdaqadaqaamaalaaabaGaeGOmaidcciGae8hWdaNaemiDaqhabaGaeGymaeJaeGOmaidaaaGaayjkaiaawMcaaaaa@387C@, C_12_ = cos⁡(2πt12)
 MathType@MTEF@5@5@+=feaafiart1ev1aaatCvAUfKttLearuWrP9MDH5MBPbIqV92AaeXatLxBI9gBaebbnrfifHhDYfgasaacH8akY=wiFfYdH8Gipec8Eeeu0xXdbba9frFj0=OqFfea0dXdd9vqai=hGuQ8kuc9pgc9s8qqaq=dirpe0xb9q8qiLsFr0=vr0=vr0dc8meaabaqaciaacaGaaeqabaqabeGadaaakeaacyGGJbWycqGGVbWBcqGGZbWCdaqadaqaamaalaaabaGaeGOmaidcciGae8hWdaNaemiDaqhabaGaeGymaeJaeGOmaidaaaGaayjkaiaawMcaaaaa@3872@, S_6_ = sin⁡(2πt6)
 MathType@MTEF@5@5@+=feaafiart1ev1aaatCvAUfKttLearuWrP9MDH5MBPbIqV92AaeXatLxBI9gBaebbnrfifHhDYfgasaacH8akY=wiFfYdH8Gipec8Eeeu0xXdbba9frFj0=OqFfea0dXdd9vqai=hGuQ8kuc9pgc9s8qqaq=dirpe0xb9q8qiLsFr0=vr0=vr0dc8meaabaqaciaacaGaaeqabaqabeGadaaakeaacyGGZbWCcqGGPbqAcqGGUbGBdaqadaqaamaalaaabaGaeGOmaidcciGae8hWdaNaemiDaqhabaGaeGOnaydaaaGaayjkaiaawMcaaaaa@3794@ and C_6_ = cos⁡(2πt6)
 MathType@MTEF@5@5@+=feaafiart1ev1aaatCvAUfKttLearuWrP9MDH5MBPbIqV92AaeXatLxBI9gBaebbnrfifHhDYfgasaacH8akY=wiFfYdH8Gipec8Eeeu0xXdbba9frFj0=OqFfea0dXdd9vqai=hGuQ8kuc9pgc9s8qqaq=dirpe0xb9q8qiLsFr0=vr0=vr0dc8meaabaqaciaacaGaaeqabaqabeGadaaakeaacyGGJbWycqGGVbWBcqGGZbWCdaqadaqaamaalaaabaGaeGOmaidcciGae8hWdaNaemiDaqhabaGaeGOnaydaaaGaayjkaiaawMcaaaaa@378A@

**Table 3:** Deviance information criteria (DICs) for models with a latent autocorrelation structure.

Model	Total	Total surgical	Total medical	EFE	Grass sickness	Large colon impaction	Large colon displacement	Lipoma
No seasonality, no trend	756.83	718.59	607.42	282.14	339.02	414.80	483.27	**419.58**
12-month seasonality, no trend	754.20	720.39	604.86	261.33	280.39	**391.20**	481.77	421.54
12- and 6-month seasonality, no trend	**732.29**	**708.46**	**592.65**	**258.93**	**267.09**	394.47	**459.30**	425.44

A lower DIC statistic can be considered to represent a better model

**Table 4:** Deviance information criteria (DICs) for models without a latent autocorrelation structure but with trend (Poisson GLMs).

Model	Total	Total surgical	Total medical	EFE	Grass sickness	Large colon impaction	Large colon displacement	Lipoma
No seasonality, no trend	796.18	728.37	645.77	280.79	329.39	429.29	513.58	422.95
12-month seasonality, no trend	793.62	729.59	645.22	258.15	289.65	411.25	513.64	425.78
12- and 6-month seasonality, no trend	773.29	721.87	633.73	**255.35**	277.67	414.21	500.42	429.93
No seasonality, trend	740.80	704.58	613.87	282.11	324.70	422.58	480.01	**413.20**
12-month seasonality, trend	735.36	704.83	611.45	259.15	284.20	**403.98**	478.60	415.88
12- and 6-month seasonality, trend	**717.63**	**698.06**	**601.9**	256.60	**272.58**	407.02	**466.76**	419.74

A lower DIC statistic can be considered to represent a better model.
